# New functional and structural insights from updated mutational databases for complement factor H, Factor I, membrane cofactor protein and C3

**DOI:** 10.1042/BSR20140117

**Published:** 2014-10-22

**Authors:** Elizabeth Rodriguez, Pavithra M. Rallapalli, Amy J. Osborne, Stephen J. Perkins

**Affiliations:** *Department of Structural and Molecular Biology, Darwin Building, University College London, Gower Street, London WC1E 6BT, U.K.

**Keywords:** age-related macular degeneration, alternative pathway, atypical haemolytic uraemic syndrome, complement structure, immunodeficiency diseases, mutation database, aHUS, atypical HUS, AMD, age-related macular degeneration, AP, alternative pathway, CFH, complement factor H, CFI, complement factor I, CUB, C1r/C1s–Uegf–Bmp1, FIMAC, factor I-membrane attack complex, HUS, haemolytic uraemic syndrome, LDLr, low-density lipoprotein receptor, MCP, membrane cofactor protein, MG, macroglobulin, SCR, short complement regulator, SNP, single nucleotide polymorphism, SP, serine protease, SRCR, scavenger receptor cysteine-rich, TED, thioester-containing domain

## Abstract

aHUS (atypical haemolytic uraemic syndrome), AMD (age-related macular degeneration) and other diseases are associated with defective AP (alternative pathway) regulation. CFH (complement factor H), CFI (complement factor I), MCP (membrane cofactor protein) and C3 exhibited the most disease-associated genetic alterations in the AP. Our interactive structural database for these was updated with a total of 324 genetic alterations. A consensus structure for the SCR (short complement regulator) domain showed that the majority (37%) of SCR mutations occurred at its hypervariable loop and its four conserved Cys residues. Mapping 113 missense mutations onto the CFH structure showed that over half occurred in the C-terminal domains SCR-15 to -20. In particular, SCR-20 with the highest total of affected residues is associated with binding to C3d and heparin-like oligosaccharides. No clustering of 49 missense mutations in CFI was seen. In MCP, SCR-3 was the most affected by 23 missense mutations. In C3, the neighbouring thioester and MG (macroglobulin) domains exhibited most of 47 missense mutations. The mutations in the regulators CFH, CFI and MCP involve loss-of-function, whereas those for C3 involve gain-of-function. This combined update emphasizes the importance of the complement AP in inflammatory disease, clarifies the functionally important regions in these proteins, and will facilitate diagnosis and therapy.

## Abstract 2

A new compilation of 324 mutations in four major proteins from the complement alternative pathway reveals mutational hotspots in factor H and complement C3, and less so in factor I and membrane cofactor protein. Their associations with function are discussed.

## INTRODUCTION

**Figure 1:**
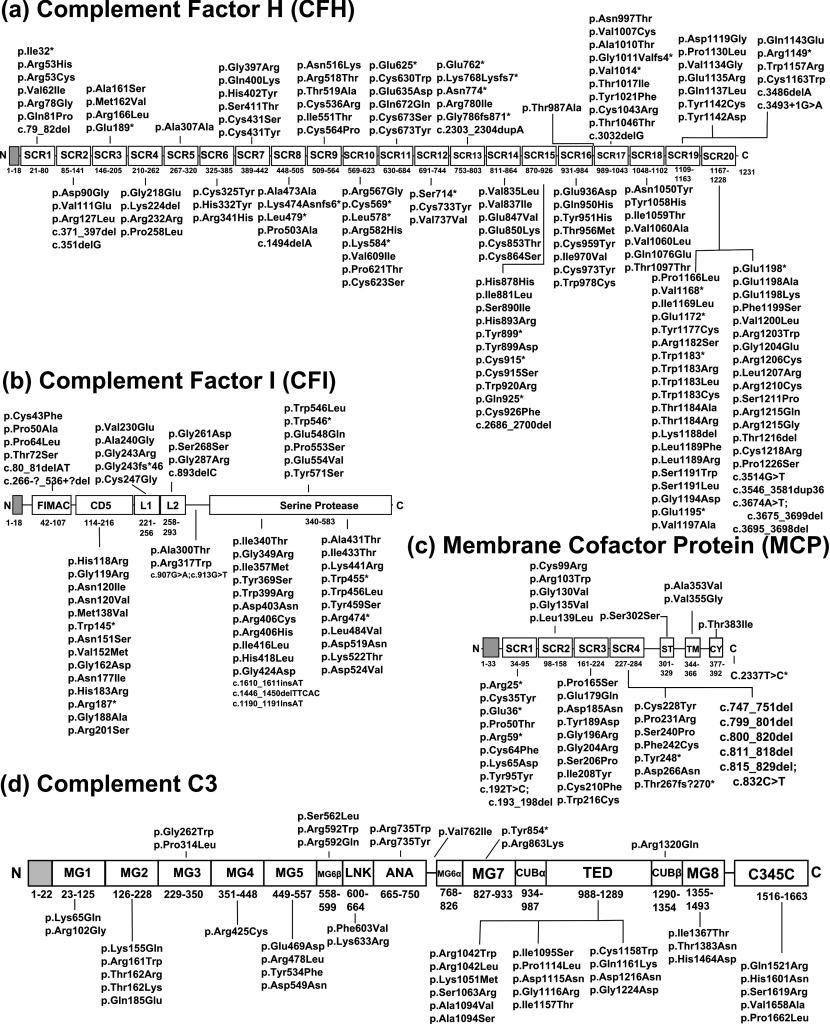
Location of 311 disease-associated mutations in the domain structures of CFH, CFI, MCP and C3 The ten intron and splicing mutations are not shown (Supplementary Table S1), making a total of 324. CFH, CFI, MCP and C3 possess 20, five, four and 13 domains respectively, which are drawn to scale. The reported sequence mutations are shown linked with the domain in which each is located. The signal peptide is shown in grey. The sequence numbering of each domain is indicated. (a) CFH with 161 mutations (SCR=short complement regulator). (b) CFI with 63 mutations (FIMAC=FI membrane attack complex; SRCR=scavenger receptor cysteine-rich; LDLr=low-density lipoprotein receptor; abbreviated as L1 and L2). (c) MCP with 41 mutations (ST=serine/threonine-rich region; TM=transmembrane helix region; Cy=cytoplasmic region). (d) C3 with 48 mutations (MG=macroglobulin; TED, thioester domain; CUB, C1r/C1s-Uegf-Bmp1 domain; and the C345C, LNK and ANA domains). Note that each of the MG6 and CUB domains is divided into two parts in the C3 sequence).

Starting from the first reports of complement disease-associated mutations [1], genetic alterations in four CFH (complement factor H), CFI (complement factor I), MCP (membrane cofactor protein) and complement C3 have increasingly been associated with immune disorders [2–4]. HUS (haemolytic uraemic syndrome) is characterized by a phenotype of microangiopathic haemolytic anaemia, thrombocytopenia and acute renal failure in affected individuals. While the majority of diarrhoeal HUS cases are caused by toxic *Escherichia coli* bacteria, other cases are non-diarrhoeal associated and have a poorer prognosis. This is termed aHUS (atypical HUS). Initially aHUS was strongly associated with mutations in the gene encoding the complement regulator CFH [5]. CFH is a central regulator in the AP (alternative pathway) of activation, in which it acts as a cofactor for the CFI in the proteolytic inactivation of the central complement protein C3b to form iC3b [6,7]. CFH also accelerates the decay of the C3 convertase C3bBb, and competes with Factor B for binding to C3b. CFH consists of 20 SCR (short complement regulator) domains, also known as short consensus repeat, complement control protein or sushi domains, each of length about 61 residues (Figure 1a).

CFI, MCP and C3 were subsequently associated with aHUS mutations. Mutations in CFH and these three other proteins result in impaired protection of host surfaces against complement activation and lead to endothelial cell activation [8]. The protease CFI is formed from a heavy chain comprising a FIMAC (FI membrane attack complex) domain, an SRCR (scavenger receptor cysteine-rich) domain (also known as a CD5 domain: cluster of differentiation 5) and two LDLr (low-density lipoprotein receptor domains [9] (Figure 1b). Its light chain is formed from the catalytic SP (serine protease) domain. CFI mutations were identified in its gene [10–12]. MCP is a widely expressed transmembrane protein that inhibits AP activation on host cells. In combination with CFI, MCP is a cofactor for the degradation of activated C3b and C4b bound to cell surfaces. The extracellular amino terminus of MCP consists of four SCR domains. Following this is an alternatively spliced region for extensive glycosylation, a transmembrane domain and a cytoplasmic domain (Figure 1c). MCP mutations were identified in its gene [11,13–16]. C3 is the most abundant complement component in plasma, occurring at about 1.0 mg/ml, and its proteolytic conversion to active C3b by the removal of its ANA domain is the central event in complement activation. C3 is formed from eight MG (macroglobulin) domains, together with the TED (thioester-containing domain) domain containing the C3b active site, the CUB (C1r/C1s–Uegf–Bmp1) domain that links the TED and MG domains, and the C345C, LNK and ANA domains (Figure 1d). On activation to C3b, the thioester forms covalent bridges with antigenic surfaces. C3 mutations were identified in its gene [17]. Mutations in other complement proteins including factor B, thrombomodulin and the CFH-related proteins also show associations with these diseases, but in low occurrences [3,18,19].

Mutational web databases provide essential repositories for functional, diagnostic and therapeutic approaches [2,20]. An interactive FH–HUS web database (http://www.FH–HUS.org) was first set up in November 2004 for mutations and structural models for CFH [5]. The update of June 2006 included mutations for CFI and MCP alongside structural models for their domains, and also included SNPs (single nucleotide polymorphisms) for these three proteins that show disease-risk associations for conditions such as age-related macular degeneration [2]. This web resource with 167 alterations received 19800 hits by June 2014. Since 2006, mutations within C3 were recognized to be important in aHUS and other diseases. Here we extend the database to include C3, and map the mutations to improved protein structural models for CFH and crystal structures for CFI, MCP and C3 [21–25]. The database now contains a new total of 324 mutations in June 2014. This enhanced dataset permits a critical review of genetic alterations in terms of function and structure. In terms of a consensus SCR structure, the distribution of 107 CFH and 22 MCP missense mutations indicate better the functional importance of the hypervariable SCR loop. The CFH mutations at its C-terminal domains are rationalized in terms of a recent co-operative two-site binding model for CFH at host cell surfaces [26]. In C3 and C3b, the proximity of the functionally important TED and MG-2 domains accounts for the highest number of mutations. The updated database provides new clarification of the role of the complement proteins in inflammatory diseases, including AMD and aHUS, and suggests new approaches to analyse the functional outcomes of such modifications.

## EXPERIMENTAL

### Mutation database updates

The FH–HUS mutation database is available at the following URL http://www.FH–HUS.org. It is installed on a server in the Department of Structural and Molecular Biology at the University College London, and backed up nightly to an off-site server. The database is constructed using mySQL (http://www.mysql.com) and is displayed and navigated on the web via PHP (http://www.php.com), Javascript and HTML programming. The database was extended to include a total of 324 mutations and new structural models for CFH, CFI, MCP and C3. Quick, basic and advanced searches can be performed for CFH, CFI, MCP and C3 mutations. Mutation maps based on both protein and nucleotide sequences are available for each protein. Statistics show the distribution of mutations in the domains of all four proteins (Figure 1). Molecular views of each missense mutation in each protein are available through the JMol java applet (http://jmol.sourceforge.net/) in order to permit a better understanding of its structural context.

### Sources of mutational data in *CFH*, *CFI*, *MCP* and *C3*

All the genetic alterations within the FH–HUS database are numbered according to the HGVS (http://www.hgvs.org/mutnomen/) guidelines for mutation nomenclature [27], where nucleotide +1 refers to the A of the initiating ATG codon. Amino acids are also numbered starting from the initiating Met residue. To avoid confusion, the website now includes a description of how the alteration was first reported if the original numbering was not consistent with HGVS guidelines.

Rare genetic alterations in populations that are thought to be the major cause of disease or deficiency are termed disease-associated mutations. SNPs are defined as point mutations that occur in over 1% of the general population. To avoid confusion, SNPs that are not associated with disease are termed non-disease causing polymorphisms, and SNPs associated with a disease risk are referred to as disease-associated polymorphisms. The 167 genetic alterations in CFH, CFI and MCP from previous [2] were retained in this database update. Additional mutations were identified from surveys of the literature database PubMed (http://www.ncbi.nih.gov/entrez/query.fcgi), and are referenced in Supplementary Table S1 [28–70]. Additional mutations were also identified by searching the Human Gene Mutation database (http://archive.uwcm.ac.uk/uwcm/mg/hgmd0.html). The database search was also extended to include records from the SNP database (http://www.ncbi.nlm.nih.gov/projects/SNP/). This resulted in 324 mutations.

**Table 1:** Summary of genetic and protein identifiers for CFH, CFI, MCP and C3 UniProt, Universal Protein Resource; OMIM, Online Mendelian Inheritance in Man (http://www.omim.org/). The reference sequences for the protein and genetic sequences are taken from the RefSeq database with the following accession numbers NM_000186.2 (*CFH*), NM_000204.2 (*CFI*), NM_002389.4 (*MCP*) and NM_000064.2 (*C3*).

Name	Gene	Chromosome location	UniProt identifier	Protein product	Amino acid length	Domain organization	Genetic disorder	OMIM identifier
CFH	*CFH*	1q31.3	P08603	Complement factor H (OMIM 134370)	1231	SCR-1 to SCR-20	Basal laminar drusen	126700
							Complement factor H deficiency	609814
							AHUS1	235400
							Macular degeneration, age-related, 4	610698
CFI	*CFI*	4q25	P05156	Complement factor I (OMIM 217030)	583	FIMAC, SRCR, LDLR-1, LDLR-2, SP	Complement factor I deficiency	610984
							AHUS3	612923
							Macular degeneration, age-related, 13	615439
							Partial deficiency of Complement C4	120790
MCP	*CD46*	1q32.2	P15529	Membrane cofactor protein (OMIM 120920)	392	SCR-1 to SCR-4	Haemolytic uremic syndrome, atypical, 2 (AHUS2)	612922
C3	*C3*	19p13.3	P01024	Complement C3 (OMIM 120700)	1663	MG1-MG8, ANA, TED, CUB, C345C, LNK	C3 deficiencyMacular degeneration, age-related, 9	613779611378
							AHUS5	612925

### Structural models for the consensus SCR domain, CFH, CFI, MCP and C3

**Figure 2:**
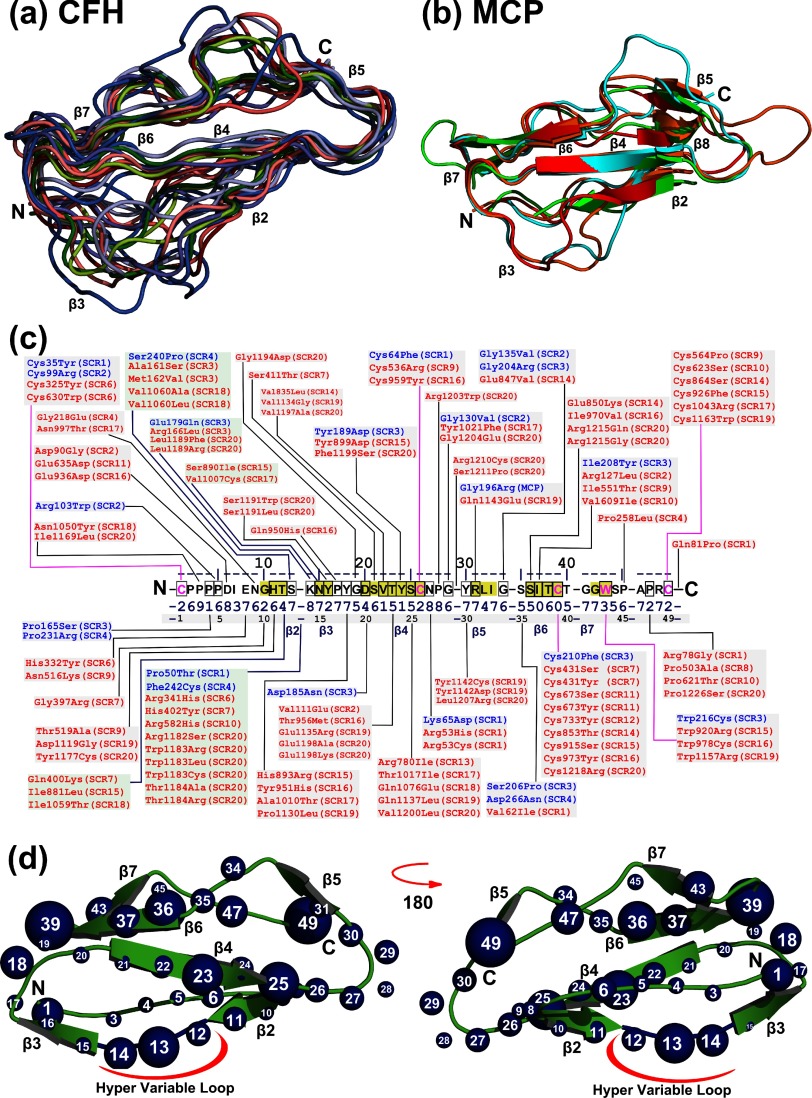
Location of CFH and MCP mutations on a consensus SCR domain The CFH and MCP missense mutations are usefully represented in terms of a consensus sequence and structure for a single SCR domain. (a) Superimposition of crystal structures available for ten SCR domains in CFH, shown as a secondary structure cartoon tube (Methods). The locations of six β-strands β2–β7 were labelled, together with the N-terminus (N) and C-terminus (C). The superimposition utilised the conserved four Cys and one Trp residues within each SCR domain, except for SCR-10 which does not contain this conserved Trp residue. (b) Superimposition of the four SCR domains in MCP, shown as a secondary structure cartoon, in the same orientation as (a). The superimposition was performed as in (a). The locations of six observed β-strands β2 and β4–β8 were labelled. (c) The consensus SCR sequence shows the most commonly occurring residue at each position. The available 106 CFH (red) and 22 MCP (blue) missense mutations are mapped to their positions within the consensus sequence. The 50 intron, deletion and silent mutations and five SCR linker missense mutations in CFH are not shown. The 21 intron, deletion and silent mutations and four non-SCR missense mutations in MCP are likewise not shown. Where mutations align with insertions outside the consensus structure, the mutation is mapped to the position before the insertion. The six main β-strands β2 to β7 in SCR domains are shown in yellow as labelled. Mutations for the five residues 11–15 in the hypervariable loop between β2 and β3 are shown against a green background. The consensus solvent side-chain accessibilities are numbered from 0 to 9, where 0 denotes 0–9% accessibility, 1 denotes 10–19% accessibility, etc. Residue side chains with values of 0 and 1 are considered to be buried. (d) Front and back view of a secondary structure cartoon derived from the consensus SCR structure. The white numbers in the spheres correspond to the residue position in the consensus sequence. Mutations are highlighted as blue spheres. The size of the individual spheres indicates the number of mutations at that position, and ranges from one to nine.

SCR domains generally contain about 60 residues arranged as six to eight β-strands numbered β1 to β8 with a hypervariable sequence loop between strands β2 and β3 (Figure 2) [2]. The consensus sequence from 124 human complement SCR sequences is shown in Figure 2(c). A total of 27 unique experimental SCR structures were superimposed to model a consensus SCR structure (Figure 2d) as previous that incorporated the six central β-strands β2–β7. The N-terminal strand (β1) and the C-terminal strand (β8) were missing from this because of the absence of residues preceding the first and last cysteine of the SCR domain. The averaged side-chain accessibility of the 27 experimental SCR structures aligned to the consensus structure is also shown in Figure 2(c). These structural information were computed from DSSP software for secondary structure assignments and side-chain surface accessibilities [71].

For full-length CFH, two structures determined by scattering modelling were used. One showed a more compact N-terminal region and more extended C-terminal region (PDB code 3GAV) [22]. The other showed a more extended N-terminal region and a more compact C-terminal region [23]. For the 20 individual domains in CFH, a total of 17 experimental structures were currently known. These were SCR-1/4 (PDB code 2WII), SCR-5, SCR-6/8 (PDB code 2V8E), SCR-10/11 (PDB code 4B2R), SCR-11/12 (PDB code 4B2S), SCR-12/13 (PDB code 2KMS), SCR-15/16 (PDB code 1HFI), SCR-18/20 (PDB code 3SW0) and SCR-19/20 (PDB code 2G7I) [72–79]. The seven structures for SCR-5, SCR-10/13 and SCR-15/16 were determined by NMR; the other ten were crystal structures which were considered to be more accurate. Previously, for the 2006 database, only five structures were known and thus used, with the other 15 domains being represented by homology models [2,5]. For the present study, the homology models from previous for SCR-9, SCR-14 and SCR17 were retained.

Crystal structures were used for CFI, MCP and C3. The structure of the five domains in CFI was represented by its crystal structure (PDB code 2XRC) [25]. This replaced the five separate homology models used previously [2]. The structure of the SCR-1/4 domains in MCP was taken from its crystal structure (PDB code 3O8E) [24]. The MCP crystal structure superseded NMR structures previously used for SCR-1 and SCR-2 of MCP, and homology models for SCR-3/4 [2,80]. The structure of the 13 domains in C3 was taken from its crystal structure (PDB code 2A73) [21].

## RESULTS

### Missense mutations within SCR domains

The SCR domain is the most abundant domain type in the complement proteins, and both CFH and MCP are entirely composed of SCR domains (Figures 1a and 1c). All 17 structures for CFH were superimposed on each other using their five conserved Cys and Trp residues. The secondary structures of SCR-1/4, SCR-6/8, SCR-10/12 and SCR-18/19 were well matched against each other, apart from minor deviations seen for SCR-6/7. However, the SCR-13 and SCR-20 structures deviated notably from this superimposed view. Crystal structures for SCR-6/7 showed similar β-strands, as also the NMR structures for SCR-5 and SCR-15/16. In the ten best-matched SCR structures, the six superimposed β-strands β2–β7 were readily visible (Figure 2a). The superimposition of the four SCR-1/4 domains in MCP also showed good matches with each other (Figure 2b), in which the β-strands β2 and β4–β8 were readily visible. Molecular graphics views showed that the best-conserved secondary structure occurred in the four-stranded anti-parallel β-sheet formed from β-strands β2, β4, β6 and β7. The four conserved Cys residues were bridged in the order C1–C3 and C2–C4 (residue pairs 1–39 and 26–49 in the consensus sequence). Since C2 occurred on β-strand β2 and C3 occurred on β-strand β6, the two disulphide bridges were crucial for the structural integrity of the SCR domain. The two-stranded anti-parallel β-sheet β5 and β8 also showed good conservation. It was relevant that all six β-strands β3–β8 were successfully identified from secondary structure predictions prior to the first SCR structure determinations, together with the partial identification of β-strand β2 [2,81]. This success indicated the degree to which the β-sheet structure in SCR domains is well-defined. The most structural variability between SCR domains occurred between the β-strands β2 and β3 at the bottom of Figures 2(a) and 2(b) (which has been termed the hypervariable loop for reason of its sequence diversity), together with partial variability between the β-strands β5 and β6 at the top of Figures 2(a) and 2(b).

Disorders in plasma proteins have been classified into two types [5,20]. Type I was defined when the protein plasma level was low and indicated a secretory defect or rapid degradation. Type II mutations were associated with normal plasma levels, being defined to correspond to a functional defect in activity. For CFH, these assignments were not straightforward for reasons of the large variation of FH levels in plasma and the different methods used for the clinical assays. We thus followed our previous analysis [2,5]. The comparison of the CFH and MCP mutational phenotypes with their protein structures was facilitated by the use of a consensus SCR domain. Such a consensus SCR structure and sequence displayed all six β-strands β2–β7 (Figure 2c) [2]. All the 107 CFH and 22 MCP missense mutations occurred within the 24 SCR domains; only one was found in a CFH inter-SCR linker between two domains (Figure 1a). This outcome suggested that the linker regions have low functional significance.

Two groups of SCR mutations attracted attention. Firstly, 25 of the 129 mutations (19%) occurred in the five residues of the hypervariable loop between β-strands β2 and β3. In contrast, the corresponding loop between the β-strands β5 and β6 only contained eight mutations. Most of these 25 mutations showed Type II phenotypes [2], and all were surface-exposed. This suggested that these mutations were associated with functional interactions. Secondly, 23 of the 129 mutations (18%) affected one of the four Cys residues that stabilized the central β-sheet. Most of these 23 mutations showed Type I phenotypes [2], and all had low-solvent exposures. This suggested that these Cys mutations were associated with structural instabilities and incorrect protein folding and expression levels.

The remaining 81 (63%) of the 129 mutations were distributed across the remaining 41 SCR residues, with between 0 and 5 occurrences at each residue (Figure 2c). A large majority of these 41 residues (i.e. 34) were solvent-exposed, and contained 62 missense mutations. Thus most of the mutations were predicted to be Type II that probably affected functional protein–protein interactions in CFH and MCP. The remaining seven residues were buried with low accessibilities of 0–2, and were associated with 19 missense mutations; these residue changes were presumed to be Type I that affected protein folding and stability.

### Analysis of disease-associated mutations within CFH

Of the 163 mutations now known for the complement regulator CFH (Supplementary Table S1), 113 were missense mutations, 21 were nonsense, 12 were deletions (of which three were in-frame), eight were silent, five were frameshifts, two were introgenic mutations, one was an insertion and one was a splicing event. The former 2006 database contained 100 genetic alterations within CFH [2]. The current distribution of 161 non-intronic mutations (Figure 1a) gave a frequency of 13 non-intronic mutations/100 residues. The 113 missense mutations were mapped onto the CFH sequence in Supplementary Figure S1, together with their computed secondary structures and side-chain accessibility for the 17 known SCR structures and three homology models. The majority of mutations were found in SCR-20 with 39, followed by SCR-19 with 13 mutations, SCR-15 with 12 mutations, SCR-17 with ten mutations and SCR-16 with eight mutations. The disproportionately high proportion of 33% (52 out of 160 mutations) in SCR-19/20 was similar to that of 39% (33 out of 84 mutations) for SCR-19/20 in the 2006 database [2]. A disproportionately high proportion of 31 mutations (19%) in SCR-15/18 was again observed, similar to that of 20 mutations (24%) reported in 2006 for SCR-15/18. The current number of mutations in other regions of CFH, especially at SCR-1/4 and SCR-6/8, was relatively low.

**Figure 3:**
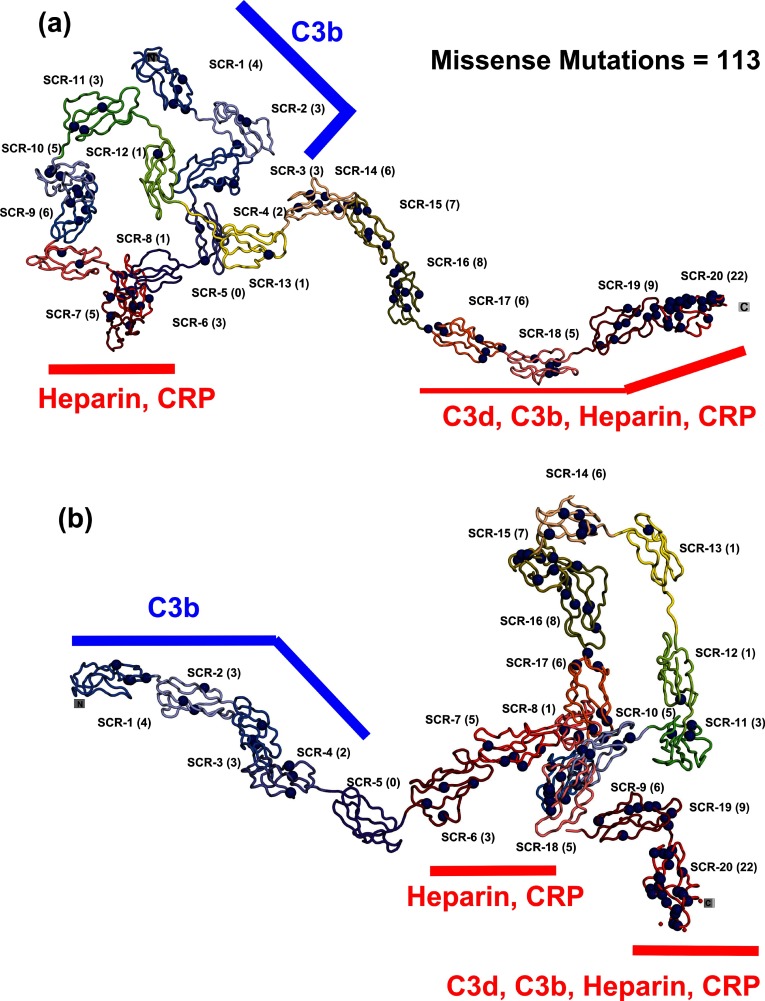
Distribution of the 100 residues associated with missense mutations in CFH The 100 residues associated with 113 missense mutations are shown as blue spheres and the number of affected residues in each SCR domain is written within brackets. The mutations are identified in Supplementary Figure S1. The 20 domain solution structure of CFH is shown as a secondary structure ribbon in two alternative views. One corresponds to a more compact N-terminal region and a more extended C-terminal region. The other corresponds to a more extended N-terminal region and a more compact C-terminal region. The scattering modelling of CFH presents these as two equivalent best-fit structures. The location of the C3b-binding site at SCR-1/4 is indicated by blue lines. The two sites for the heparin and C-reactive protein-binding sites are shown by red lines. C3b and C3d also bind at the C-terminus.

A structural understanding of the CFH mutations required a molecular structure for CFH, however intact CFH has not been crystallised to date. Fortunately, experimental structures were now known for 17 of the 20 SCR domains, with many from crystallography (Methods). Most SCR domains in CFH possessed typical SCR structures with up to eight β-strands (Figure 2a); the exceptions were SCR-13 and SCR-20. The middle SCR domains of CFH possessed shorter sequences, longer inter-domain links and higher glycosylation levels, suggesting that these middle domains acted as conformational spacers. Scattering modelling produced the first experimental molecular structures for full-length CFH [22,23]. This modelling was based on molecular structures for all 20 SCR domains joined together; conformational randomization was applied to the inter-SCR linkers to create trial CFH domain arrangements in all conformations. The scattering fits showed that only the folded-back domain structures with an overall length of 40 nm fitted the data, not the extended ones with a length of up to 73 nm. Two distinct CFH models had been determined, both giving indistinguishable scattering curve fits. In the first, SCR-13/20 is extended in its domain arrangement and SCR-1/12 is looped back (Figure 3a) [22]. In the second, the SCR-1/7 domains are extended and SCR-8/20 is looped back (Figure 3b) [23]. The second model was more easily docked with the C3b–CFH crystal structure in which the SCR-1/4 domain arrangement was extended [63]. As the SCR-6/8 and SCR-19/20 domains may potentially bind to a surface, both CFH structures were consistent with the recently–proposed bivalent and cooperative binding mechanism of CFH to polyanionic oligosaccharides on a host cell surface [26,82].

CFH possessed major functional activities at its N-terminal SCR-1/4 and C-terminal SCR-19/20 domains, together with another activity at the SCR-6/8 domains [83]. The mapping of the 112 CFH missense mutations onto 100 mutated residues showed that the mutations occurred throughout the CFH structure. The availability of 17 experimental SCR structures for CFH means that side-chain solvent accessibilities were more precisely estimated. Inspection of these accessibilities for the CFH mutations showed that 67 out of 100 mutated residues were solvent exposed, while 33 out of 100 corresponded to buried side chains with low-solvent accessibilities of 0–2 (Supplementary Figure S2).

Mutations were not evenly distributed in CFH. The mutational hotspot at SCR-19/20 showed 22 solvent-exposed sidechains in 31 mutated residues. The frequency of non-intronic mutations/100 residues rose from 13 for full-length CFH to 44 in SCR-19/20. This abundance in SCR-19/20 correlated with the presence of key binding sites for C3b, C3d and heparin-like ligands. The best understood were the two binding sites for C3d on SCR-19/20, one on each domain [84,85], which may account for the high proportion of mutations in SCR-19/20. C-reactive protein was also reported to bind to SCR-16/20 [86]. The proportionately higher number of SCR-15/17 mutations implied that SCR-15/17 may be functionally significant, even though these did not contain reported ligand-binding sites. The folded-back domain arrangement of SCR-15/17 in the second CFH model (Figure 3b) suggested that this defined the separation of the two heparin-like binding sites at SCR-6/8 and SCR-19/20 in the bivalent binding of CFH to host cell surfaces. If so, mutations in SCR-15/17 would alter the separation between these two heparin sites and disrupt CFH binding to host cell surfaces. Curiously, despite the well-characterized binding of SCR-1/4 to C3b [72] and that of heparin and C-reactive protein to SCR-6/8 [82,86], fewer mutations occurred in the SCR-1/4 and the SCR-6/8 regions.

### Analysis of disease-associated mutations within CFI

Of the 64 mutations currently known for the regulatory protease CFI (Supplementary Table S1), 49 were missense mutations, six were nonsense, two were deletions, one was silent, one was a frameshift, two were insertions and one was a splicing event. The distribution of the 63 non-intronic mutations was summarized in Figure 1(b). The 49 missense mutations were mapped onto the CFI sequence in Supplementary Figure S2, together with their computed secondary structures and side-chain accessibilities. The former 2006 database had 24 genetic alterations within CFI, with only seven missense mutations and three nonsense mutations [2]. The present database identified 49 missense mutations in all five domains of CFI. Although most (18) were within the SP domain, this was as expected because the SP domain was the biggest one. All five domains showed similar mutational frequencies of 6–14 non-intronic mutations/100 residues. Unlike CFH, no sequence mutational hotspots or unaffected parts of CFI occurred that might reflect different functional activity.

**Figure 4:**
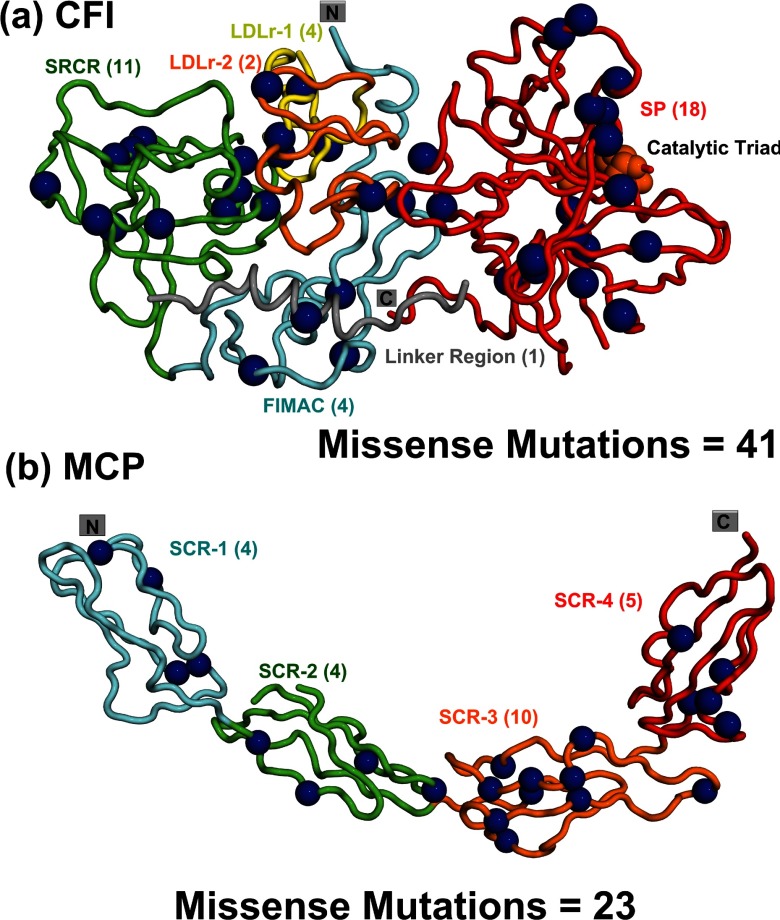
Distribution of the CFI and MCP missense mutations in their crystal structures The domains are shown as a secondary structure ribbon, coloured from blue (N-terminus) to red (C-terminus) in a rainbow scheme. The number of mutations within each domain is written within brackets, and the mutations are identified in Supplementary Figures S2 and S3. (a) Location of 40 residues associated with 41 missense mutations are shown as dark blue spheres within the crystal structure of CFI. Factor I is a heterodimer consisting of a non-catalytic heavy chain (FIMAC domain, blue; SRCR domain, green; LDLr-1 domain, orange; LDLr-2 domain, yellow), and linked by a disulphide bound to a catalytic light chain (SP domain, red). This colour scheme follows that for the sequence in Supplementary Figure S2. For the linker, one missense mutation is not shown for reason of disorder in the crystal structure, making a total of two. For the SP domain, seven missense mutations are not shown for reason of disorder in the crystal structure, making a total of 25. The catalytic triad residues in the SP domain are shown as orange spheres. (b) Location of 23 residues associated with 23 missense mutations within the crystal structure of the extracellular domains of MCP. Mutations are mostly found in the four extracellular SCR domains (SCR-1, cyan; SCR-2, green; SCR-3, orange; SCR-4, red). This colour scheme follows that for the sequence in Supplementary Figure S3.

The CFI crystal structure located the residues in CFI, excepting those not visible through protein disorder at surface loops and its compact domain arrangement [25]. The compact domain structure (Figure 4a) was similar to that first established by solution scattering modelling [9]. Compared with the five separate domain structures used previously [2], the crystal structure provided accurate solvent side-chain accessibilities. Thus 19 out of 48 missense mutations corresponded to buried side chains with low-solvent accessibilities of 0–2 (Supplementary Figure S2). This proportion of 40% buried mutated residues in CFI was higher than those of 23% buried mutated residues in the consensus SCR and 33% for the buried mutated residues in CFH. This 40% proportion was attributed to the compact CFI structure, meaning that mutations may perturb this arrangement, compared to the more extended structures of CFH and MCP. Although several CFI missense mutations were close to the catalytic triad (Figure 4a), no mutational clustering was seen in the CFI structure.

### Analysis of disease-associated mutations within MCP

Of the 49 mutations presently known for MCP (Supplementary Table S1), 26 were missense mutations, four were nonsense, six were deletions, three were silent, one was a frameshift, six were introgenic mutations and three were polymorphisms. The total of 26 missense mutations is increased compared to ten previously [2]. The distribution of 43 non-intronic mutations was summarized in Figure 1(c). The four extracellular SCR domains showed similar mutational frequencies of 15 non-intronic mutations/100 residues. The 26 missense mutations were mapped onto the MCP sequence in Supplementary Figure S3, together with their secondary structures and side-chain accessibility. The most missense mutations occurred in SCR-3; this abundance may be correlated with surface exposed residues associated with both C3b and C4b binding to MCP [87].

The MCP crystal structure (Figure 4b) revealed an extended four-domain arrangement [24]. Of the 23 missense mutations, 13 were located at solvent-accessible sidechains, while ten corresponded to buried sidechains with low solvent accessibilities of 0–2 (Supplementary Figure S3). MCP showed a higher proportion (43%) of buried missense mutated side chains than CFH (33%). This increased proportion was attributed to the importance of the four Cys residues in stabilizing the β-sheet SCR structure. Five of these ten buried residues in MCP occurred at conserved Cys residues.

### Analysis of disease-associated mutations within C3

Of the 48 mutations presently known for C3 (Supplementary Table S1), 47 were missense mutations, and one was a nonsense mutation. No significant number of C3 mutations was known previously [2]. The 47 missense mutations were mapped onto the C3 sequence in Supplementary Figure S4, together with their secondary structures and side-chain accessibilities. The most missense mutations were found in the TED domain with 15, followed by the MG2 and MG5 domains with 4–5 in each, and the C345C domain with four (Figure 1d). The mutational frequencies in the TED, MG2 and MG5 domains were five non-intronic mutations/100 residues, that in the ANA domain was 3.5 non-intronic mutations/100 residues, and that in the C345C domain was 2.7 non-intronic mutations/100 residues. These mutational frequencies were less than half of those in CFH, CFI and MCP.

**Figure 5:**
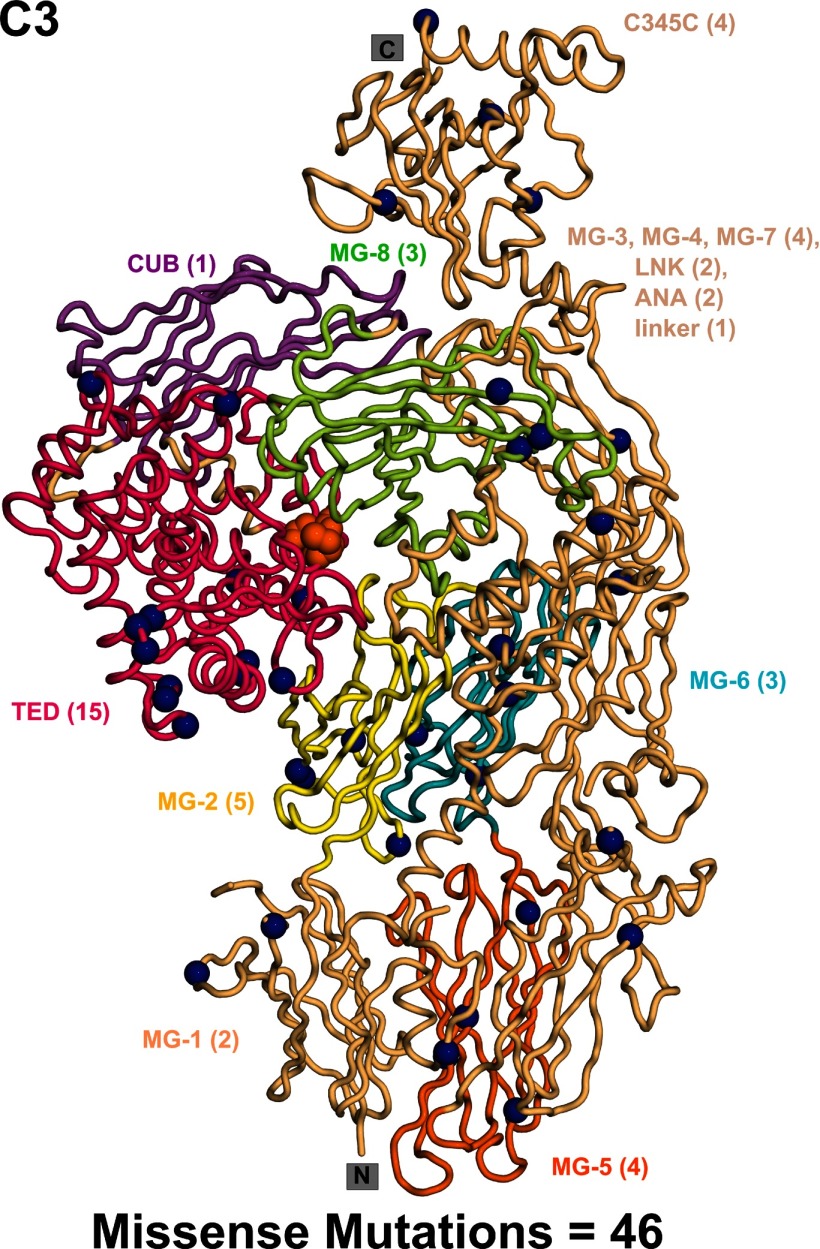
Distribution of the C3 missense mutations in the C3 crystal structure The domains within the crystal structure of C3 are shown as a secondary structure ribbon. The functionally important TED domain is shown in bright pink, and the adjacent CUB domain is shown in dark pink. The location of the thioester residues in the TED domain are shown as orange spheres. The MG1–MG8, C345C, LNK and ANA domains are shown in light orange, except for the MG2 domain (yellow), MG5 domain (orange), MG-6 (blue) and MG8 domain (pale green) because these four MG domains possess greater numbers of mutations. This colour scheme mostly follows that for the sequence in Supplementary Figure S4. The 45 residues associated with 47 missense mutations are shown as dark blue spheres. The number of mutations within each domain is written within brackets, and the mutations are identified in Supplementary Figure S4.

**Figure 6:**
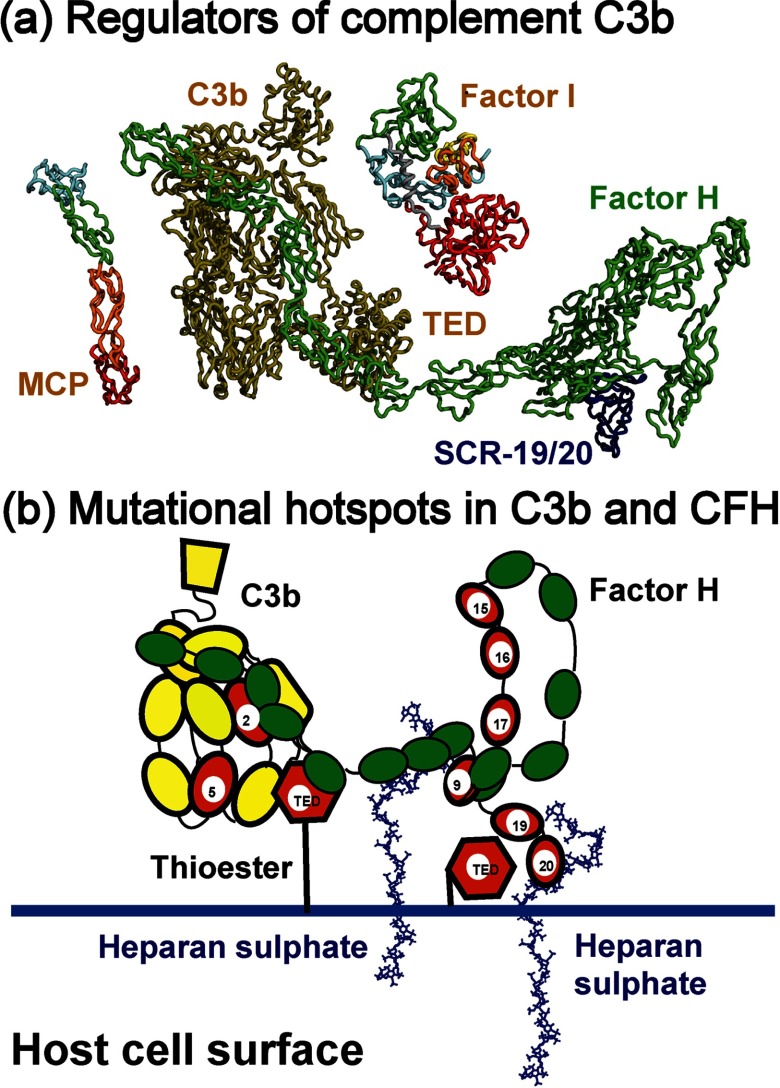
Schematic views of CFH and C3b binding to a host cell surface (a) CFH is represented by a green cartoon tube showing the 20 SCR domains, with SCR-19/20 highlighted in blue. The SCR-1/4 domains are shown bound to C3b in yellow. In C3b, unlike in C3, the TED domain is extended to the base of the C3b structure. The CFI and MCP regulators are drawn to the same scale using the same colouring of Figure 4. The SP domain of CFI cleaves the CUB domain of C3b with CFH acting as cofactor. (b) The CFH–C3b complex is shown as a cartoon in the same orientation as in (a). CFH binds bivalently at its SCR-7 and SCR-20 domains to anionic charges on the heparan sulphate-coated host cell surface (represented by heparin dp36). CFH also binds to covalently linked C3d (TED) on host cell surfaces. This bivalent binding positions the SCR-1/4 domains in a conformation that binds readily to C3b when C3b is covalently bound to host cell surfaces through its thioester bond in the TED domain. The C3b interaction with CFH results in the CFI-mediated degradation of C3b to form C3d (the TED domain) and C3c, thereby regulating complement activation at host cell surfaces. The TED, MG2 and MG5 domains showing the most C3b mutations are highlighted in red. The SCR-19/20 domains that interact with C3d also possess high levels of CFH mutations, also in red. The SCR-9 and SCR-15/17 domains that act as spacers of the SCR-6/8 and SCR-19/20 domains also possess high levels of mutations, and are indicated in red.

The C3 crystal structure with all 13 domains was used for mutational analyses (Figure 5) [21]. The ANA domain was removed in activated C3b, thus this crystal structure was not used, but was made available on our updated web site at http://www.FH–HUS.org. In the C3b crystal structure, which was crystallized in low-salt conditions, the TED and CUB domains (red and purple in Figure 5) moved downwards to form a TED–MG1 domain pair (Figure 6a). In more physiological salt buffers, the TED–MG1 domain pair in C3u and C3b became separated [88–90]. The C3 crystal structure showed the inter-domain packing arrangement, although these underwent rearrangement in C3b. Inspection of the solvent side-chain accessibilities showed that 12 out of 46 missense mutations corresponded to buried side chains with low-solvent accessibilities of 0–2 (Supplementary Figure S4). This 26% proportion of buried missense residues in C3 was the lowest compared with CFH (33%), CFI (40%) and MCP (43%). This was explained by the paucity of Cys residues in C3 compared to those of the Cys-rich CFH, CFI and MCP structures, thus making the C3 structure less vulnerable to mutational losses of its Cys residues.

The C3 mutations could be correlated with structure–function relationships. The higher number of TED, MG2 and MG5 mutations coincided with the location of the main CFH-binding site between the TED–CUB domains and the MG1–MG2 domains in the crystal structure of the C3b and CFH SCR-1/4 complex [72]. The regulatory breakdown of C3b depended on the correct relative positioning of these TED–CUB and MG1–MG2 domains (Figure 5). In addition, the thioester bridge of the TED domain binds to pathogenic targets, thus the TED missense mutations may reflect its interactions with pathogens (Figure 5). The TED domain also binds to complement receptors types 1 and 2 at different contact sites, and these may be affected by missense mutations in the TED domain [92].

The Arg102Gly missense mutation on the MG1 domain defined a polymorphism that distinguished the major C3S and C3F allotypes of C3. C3S was the most common of the two with frequencies of 0.79 and 0.99 in Caucasian and Asian populations, respectively. The functional polymorphism C3F was also identified as the rs2230199 SNP. C3S and C3F were distinguished from their electrophoretic mobility on agarose gels, with C3S moving slower and C3F faster [78]. Because the C3b crystal structure showed that Arg102 on the MG1 domain was in proximity to negatively charged TED residues, the Arg102Gly change weakened the interaction of the TED–MG1 domain pair [54,90].

## DISCUSSION

In this study, the doubling of the available mutations in the FH–HUS database, together with an almost complete set of crystal structures for CFH, CFI, MCP and C3/C3b, provided novel insight on the involvement of CFH, CFI, MCP and C3 with aHUS, AMD and other inflammatory diseases. These four proteins are crucial for the correct functioning of the AP, summarized in Figure 6(a). The combination of the mutations with structures permits more accurate assessments of the consequence of a residue change in an integrated view of complement activation and its regulation. The complement system plays an important role in the pathogenesis of aHUS and AMD, with C3/C3b being central to this. Because all three complement activation pathways converge at C3, the regulation of activated C3b is tightly controlled by CFH or MCP as cofactor and CFI as protease. This would account for the susceptibility of these four proteins to genetic alterations. If complement is inefficiently regulated, host cells become subject to complement attack, resulting in tissue damage and a spectrum of complement-related diseases. CFH was the first protein to be associated with genetic disease, followed by CFI and MCP, and then by C3/C3b. The number of mutations currently corresponds to totals of 163 in CFH, 64 in CFI, 49 in MCP and 48 in C3. Of these, 127 CFH mutations, 30 CFI mutations, 48 MCP mutations and 36 C3 mutations were linked with aHUS patients. The non-intronic mutational frequencies in these four proteins are 13 for CFH, ten for CFI, nine for MCP and three for C3/C3b, each normalized to 100 residues. Other complement proteins show much reduced mutational frequencies [3,32].

Although single mutations in CFH and C3 were enough to predispose for aHUS, a low proportion of aHUS patients with MCP or CFI mutations presented mutations in other complement proteins [32]. The CFH, CFI and MCP mutations have been discussed previously [2]. Since that 2007 study, many new C3 mutations have been identified. Patients with point mutations at a 5′ donor splice site in the C3 gene produce dysfunctional C3 protein, and show heightened susceptibility to bacterial infections. The R1320Q mutation in the CUB domain occurs at one of the two Arg–Ser sequences, which is cleaved by CFI, leading to resistance to CFI-mediated cleavage [57]. Several C3 mutations have been implicated in AMD and aHUS, including K65Q, K155Q, R161W, P314L and R102G. For example, the K65Q variant in the MG1 domain causes decreased binding of CFH to C3b [52], leading to inefficient complement inactivation and endothelial damage to the glomeruli. The K155Q alteration in the MG2 domain shows a ~2.9-fold increased risk of AMD [56]. The K155Q variant in C3 causes reduced CFH binding to C3b, thus inhibiting the ability of CFH to regulate C3b. The R161W variant in C3 causes hyperactive C3 convertase activity; this change increased the binding of C3b to factor B and reduced the binding of C3b to CFH and MCP, and resulted in a rapid progression to end stage renal failure [57]. Interestingly, these C3 variants involve replacements of positively charged residues. Mutations in C3 often lead to the reduced binding of CFH and MCP to the mutant C3b which is then less effectively regulated. Other C3 mutants cause C3b to bind more strongly to factor B, resulting in increased C3 convertase formation. Both scenarios correspond to a ‘gain of function’ in C3 and C3b. In distinction to these C3 mutations, mutations in CFH, CFI and MCP correspond to a ‘loss of function’ in these three proteins, meaning also that C3b is less regulated and exhibits an increase in activity, thus leading to inflammation.

Mutations were generally explained in terms of their functional consequences, although these explanations are not necessarily definitive and some will require experimental validation. For example, the R102G polymorphism is strongly associated with aHUS and AMD [54], yet its function is unknown, and the database will facilitate the design of experiments to probe this. Comparison of the two binding sites for C3d on SCR-19/20 showed that three mutations in C3d and seven mutations in SCR-19/20 are associated with the contact residues [84,85]. These can likewise be tested through mutagenesis. Some anticipated mutational hotspots were not observed in functional regions. However this outcome has been seen with other proteins. For example, 14000 mutations were reported for the tumour suppression protein p53, but the effect of only about half of these could be explained [91]. The structural analysis of 1119 unique haemophilia B mutations in the 415-residue coagulation protease factor IX (including 604 missense mutations) revealed notably fewer mutations in the Gla domain compared with the other three domains, even though the membrane-binding Gla domain is essential for function in factor IX [20].

Mutational hotspots were primarily identified in CFH and C3/C3b (Figure 6b). For CFH, mutational hotspots were observed for the SCR-19/20 and SCR-15/18 domains, but less so for the SCR-1/4 and SCR-6/8 domains (Figure 6b). The SCR-19/20 mutations may correlate with functional binding sites for C3b/C3d and heparin. In the case of SCR-15/18, these domains separate the two heparin-binding sites at SCR-6/8 and SCR-19/20, thus mutations may affect the bivalent binding of CFH to heparan sulphate-coated host cells [26]. Also C-reactive protein binds to SCR-16/20 and may be implicated with mutations in this region [86]. Comparatively fewer CFH mutations were observed for SCR-1/4 and SCR-6/8 even though these bind to C3b and heparin, respectively. For MCP, the mutations in SCR-1/4 may relate with MCP binding to C3b/C4b, similar to that of CFH SCR-1/4. For CFI, which has a different domain structure to those of CFH and MCP (Figure 4a), the broader occurrence of mutations in its structure may be related to extensive interactions of its SP domain with the CUB domain in C3b, and its SRCR domain with the C345C domain in C3b (Figure 6a). C3/C3b differs in that many of its 13 domains are not disulphide-rich and domain rearrangements occur when C3 is activated to C3b. A given mutation may affect either C3 or C3b or both. The most affected region in C3/C3b is the TED domain that contains the reactive thioester group, followed by the nearby MG2 and MG5 domains (Figure 5). This mutational hotspot is explained by the multiple roles of the TED domain. TED interacts covalently with antigenic targets, as well as with the C-terminus of CFH and also with complement receptors 1 and 2. These multiple interactions may lead to the greater number of mutations observed in TED.

The updated database will assist immunologists to cross-correlate mutations detected in these four different proteins for a given patient. For example, for AMD patients, the database highlights the risk factors associated with associated polymorphisms, and whether or not there are any links with aHUS. A clearer definition of aHUS will assist the design of treatment strategies, including decisions on transplants. The four proteins CFH, CFI, MCP and C3/C3b show different patterns of mutational change. The Type I phenotype corresponds to low protein levels caused by misfolding or degradation events, whereas Type II phenotypes correspond to the loss of functional activity in normal levels of the protein. How individual CFH mutations may result in the observed Type I and II phenotypes was previously discussed [2]. The larger number of CFH and MCP mutations compared with CFI and C3 support the view that Type I phenotypes often result from mutations in the four disulphide-linked Cys residues in the consensus SCR domain, which tend to be at solvent-inaccessible buried positions. Type II phenotypes may often result from alterations in residues at surface-exposed positions. In this context, the AMD-associated Tyr402His polymorphism and other type II mutations associated with aHUS were located within the hypervariable loop between β-strands β2 and β3, suggesting that this exposed loop is often involved in function.

As well as suggesting future avenues of experiment, the complement mutations will facilitate an evolutionary analysis of the complement proteins. This was illustrated by an evolution study of the coagulation proteins [94]. Complement is genetically related to coagulation. Eleven human coagulation factors were found in all vertebrates, suggesting that they emerged with the first vertebrates around 500 Mya. Sequence analyses of 47 genomes revealed that the coagulation system was under strong selective pressures, perhaps to adapt against blood-invading pathogens. Interestingly, the number of disease-causing mutations was inversely related to the probability of positive selection. When a site was under positive selection, it was less likely to acquire disease-causing mutations. In contrast, sites under negative selection were more likely to be associated with destabilizing disease-causing mutations. A limited analysis of six genomes suggested that positive selection occurred in nine complement genes [82].
